# Factors Associated With Primary Care Physician Decision-making When Making Medication Recommendations vs Surgical Referrals

**DOI:** 10.1001/jamanetworkopen.2022.56086

**Published:** 2023-02-15

**Authors:** Anusha Naik, Solomiya Syvyk, Jason Tong, Chris Wirtalla, Frances K. Barg, Carmen E. Guerra, Shivan J. Mehta, Richard Wender, Raina M. Merchant, Rachel R. Kelz

**Affiliations:** 1 Center for Surgery and Health Economics, Department of Surgery, University of Pennsylvania, Philadelphia; 2 Perelman School of Medicine, University of Pennsylvania, Philadelphia; 3 Leonard Davis Institute of Health Economics, University of Pennsylvania, Philadelphia; 4 Department of Surgery, Perelman School of Medicine, University of Pennsylvania, Philadelphia; 5 Department of Family Medicine and Community Health, Perelman School of Medicine, University of Pennsylvania, Philadelphia; 6 Department of Medicine, Perelman School of Medicine, University of Pennsylvania, Philadelphia; 7 Department of Emergency Medicine, Perelman School of Medicine, University of Pennsylvania, Philadelphia

## Abstract

**Importance:**

Although objective data are used routinely in prescription drug recommendations, it is unclear how referring physicians apply evidence when making surgeon or hospital recommendations for surgery.

**Objective:**

To compare the factors associated with the hospital or surgeon referral decision-making process with that used for prescription medication recommendations.

**Design, Setting, and Participants:**

This qualitative study comprised interviews conducted between April 26 and May 18, 2021, of a purposive sample of 21 primary care physicians from a large primary care network in the Northeast US.

**Main Outcomes and Measures:**

Main outcomes were the factors considered when making prescription medication recommendations vs referral recommendations to specific surgeons or hospitals for surgery.

**Results:**

All 21 participant primary care physicians (14 women [66.7%]) reported use of evidence-based decision support tools and patient attributes for prescription medication recommendations. In contrast, for surgeon and hospital referral recommendations, primary care physicians relied on professional experience and training, personal beliefs about surgical quality, and perceived convenience. Primary care physicians cited perceived limitations of existing data on surgical quality as a barrier to the use of such data in the process of making surgical referrals.

**Conclusions and Relevance:**

As opposed to the widespread use of objective decision support tools for guidance on medication recommendations, primary care physicians relied on subjective factors when making referrals to specific surgeons and hospitals. The findings of this study highlight the potential to improve surgical outcomes by introducing accessible, reliable data as an imperative step in the surgical referral process.

## Key Points

**Question:**

How do the factors associated with primary care physician decision-making differ for medication recommendations compared with surgical referrals?

**Findings:**

This qualitative study found that primary care physicians use evidence-based decision support tools to make medication recommendations and used professional experiences, subjective information on quality, and convenience when making surgical referrals.

**Meaning:**

This study suggests that there is an opportunity to reduce variability and improve surgical outcomes by supporting primary care referral decision-making with valid and reliable data on surgeon and hospital quality.

## Teaser

This qualitative study describes the factors primary care physicians report considering when deciding whether to offer patients prescription medication vs a surgical referral.

## Introduction

Primary care physicians (PCPs) initiate more than 51 million referrals annually.^1^ With substantial differences in practice patterns, management of the referral process is critical to facilitate advances in patient satisfaction, population health, and cost containment.^2,3^

Given the variation in quality across surgeons and hospitals in the US, referrals for high-quality surgical care provide an opportunity to improve patient care.^4^ Although data on surgeon and hospital performance are publicly available for clinicians at the time of referral decisions, it is not clear if and how such data are applied.

In an effort to identify a new mechanism to promote high-quality surgical care, we sought to understand how PCPs select specific surgeons and hospitals for referrals. We conducted a qualitative content analysis of qualitative interviews with PCPs to compare the information used to make medication prescription recommendations vs referrals to specific surgeons or hospitals. We hypothesized that PCPs could approach direct referrals to surgical care with a similar evidence-based method as other medical treatment decisions.^5^

## Methods

We designed a qualitative study of PCPs at Penn Medicine, representing more than 90 practices located in the greater Philadelphia region. Using a purposive sample of male and female PCPs (with differing races and ethnicities and levels of experience and from varying practice zip codes), 20-minute semistructured interviews, including open-ended questions and free-text lists of answers (“freelisting”), were conducted on a video platform between April 26 and May 18, 2021. Interviews were performed by a formally trained female research coordinator (S.S.) under the supervision of a medical anthropologist (F.K.B.) and the principal investigator (R.R.K.). There was no prior relationship between interviewer and participants, who were approached via email. Respondent characteristics, provided via a screening questionnaire, were obtained and summarized (eTable 1 in Supplement 1). The interview guide was developed based on prior literature,^6,7^ with input from coinvestigators specializing in primary care (C.E.G., S.J.M., and R.W.), the medical anthropologist (F.K.B.), and the principal investigator (R.R.K.). The guide contained 4 freelisting prompts and 4 open-ended questions designed to understand participants’ prescribing and surgical referral practices (eTable 2 in Supplement 1). Interviews were recorded (with oral consent from the physicians), transcribed, and deidentified by a second independent reviewer (A.N.). This study was approved by the institutional review board of the University of Pennsylvania and prepared in compliance with the Consolidated Criteria for Reporting Qualitative Research (COREQ) reporting guideline.

Freelist responses were reviewed by the study team to standardize word forms, and a salience index (the Smith *S*) was calculated for each term using the formula *S* = [(*L* − *R_j_* + 1)/*L*]/*N* with Anthropac (Analytic Technologies), where *L* is the length of each list, *R_j_* is the rank of item *j* in the list, and *N* is the number of lists in the sample.^8,9^ Salience is a function of how frequently a word is mentioned by most people early in their list. Saturation testing was performed by analyzing the freelist results when approximately 60% of the data were collected and then rerunning analyses when sampling was finished. No new words or concepts arose during this interval.^10^

An integrated approach was used to create a codebook; an a priori set of codes was derived from the freelisting results, and inductive codes were derived from the interviews.^11,12^ Three members of the study team (A.N., S.S., and J.T.) analyzed the data. Inductive codes were identified by a line-by-line review of the interviews. Each code was defined, and decision rules for their application were included in their definition, resulting in a coding tree. All transcripts were coded by at least 2 members of the study team. Discrepancies in coding were adjudicated by the principal investigator (R.R.K.). Searches for discrepant cases and peer debriefing were used to ensure trustworthiness of the data interpretation. Stata, version 16.1 (StataCorp LLC) was used for population summary statistics. Anthropac, version 4.98 (Analytic Technologies) was used to analyze the freelisting data.

## Results

Data saturation was achieved after interviews with 21 PCPs (14 women [66.7%] and 7 men [33.3%]). Participant demographic characteristics are displayed in detail in Table 1.

**Table 1.** Demographic Characteristics of Primary Care Physician Participants

Characteristic	Participants, No. (%) (N = 21)
Gender^a^	
Female	14 (66.7)
Race^a^	
Asian American or Pacific Islander	4 (19.0)
White	13 (61.9)
Unknown	4 (19.0)
Hispanic or Latinx^a^	
Yes	1 (4.8)
Age group of patients seen most often, y^a^	
30-55	16 (76.2)
56-69	5 (23.8)
Primary specialty	
Family medicine	10 (47.6)
Internal medicine	11 (52.4)
Years in practice	
≤10	9 (42.9)
11-29	9 (42.9)
≥30	3 (14.3)
No. of partners in practice	
1-3	4 (19.0)
4-9	3 (14.3)
≥10	14 (66.7)
Use of electronic health record	
Yes	21 (100)
Minority-serving practice	
Yes	19 (90.5)
Not sure	1 (4.8)
Unknown	1 (4.8)
Geriatric-serving practice	
Yes	19 (90.5)
No	1 (4.8)
Not sure	1 (4.8)
Practice in urban setting	
Yes	16 (76.2)
No	3 (14.3)
Unknown	2 (9.5)
University-affiliated practice	
Yes	19 (90.5)
No	1 (4.8)
Unknown	1 (4.8)
Hospital-affiliated practice	
Yes	19 (90.5)
Not sure	2 (9.5)

^a^ Response was optional.

Freelist responses demonstrated that, when deciding whether to prescribe a specific medication within a given clinical indication, PCPs relied predominantly on decision support tools (DSTs) with the consideration of patient attributes. When differentiating between specific surgeons or hospitals for surgical referrals, salient freelisting responses included the participants’ own professional training and experiences, perceived quality of surgical care, and perceived convenience. Although the existence of referral-related DSTs was acknowledged, their application in the surgical referral process was not mentioned.

During the semistructured interviews, PCPs elaborated on their freelisting responses by providing further details, thus building on the 3 salient terms (Figure). The concept of professional experience varied with years in practice. Newer PCPs reported using recommendations from senior PCPs and trust in the institution to hire highly competent surgeons. Senior PCPs reported incorporating patient feedback and prior interactions with specialists to inform future referrals. Primary care physicians defined surgeon quality by review of outcomes, volume, and reputation. However, on probing, PCPs recounted the absence of objective data on outcomes, resulting in a reliance on anecdotal interactions, including word-of-mouth reputation, as a proxy for quality. Bedside manner, care coordination, and availability were also considered components of quality. Limitations of existing data on surgical quality are listed in Table 2.

**Figure.**
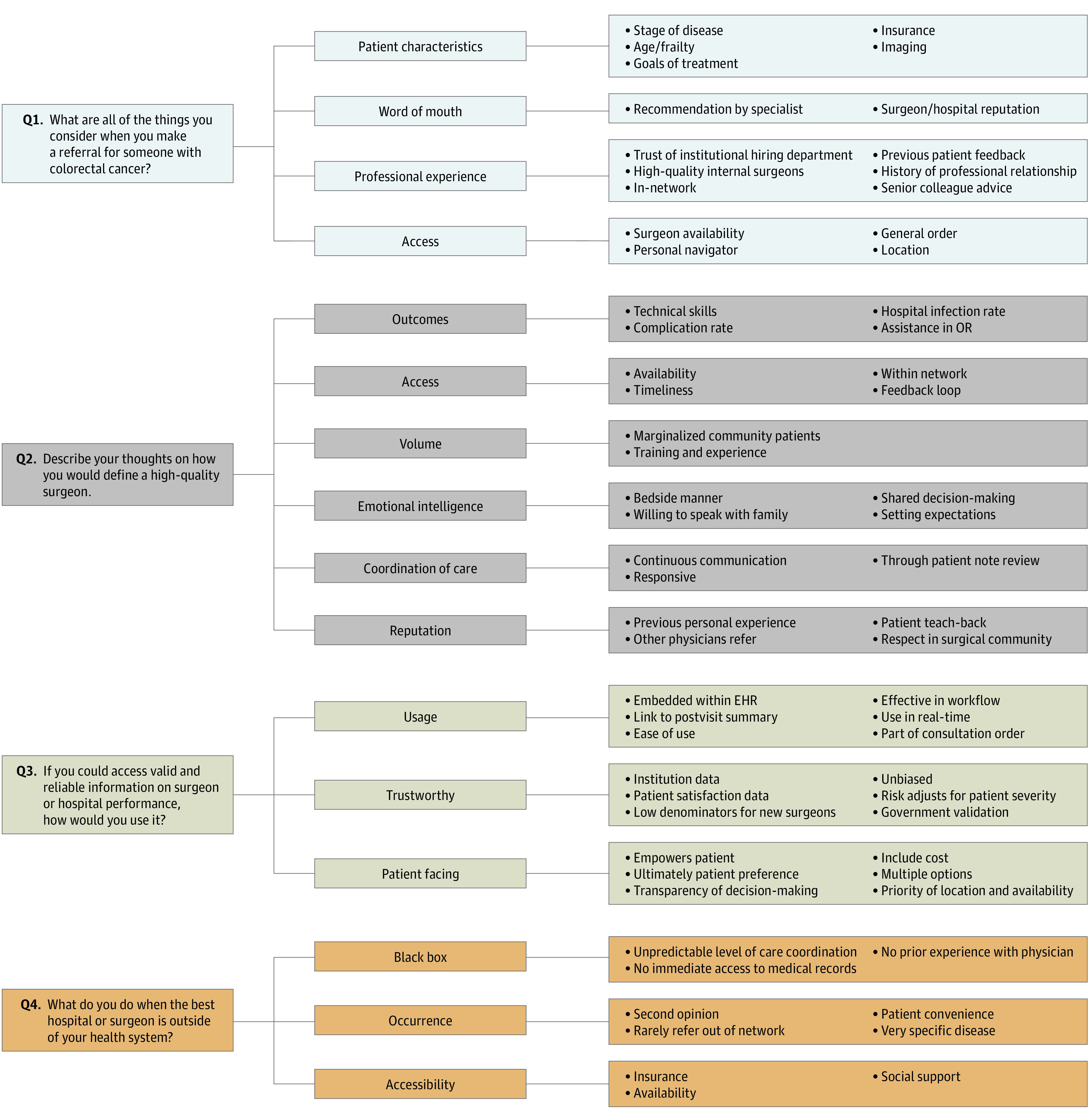
Coding Tree Derived From Open-Ended Interviews EHR indicates electronic health record; OR, operating room.

**Table 2.** Primary Care Physician Quotations that Identify Potential Data Sources Available to Facilitate Surgical Referrals and Their Associated Limitations^a^

Data source	Limitation	Example quote
Centers for Medicare & Medicaid Services website; ProPublica	Unclear data quality	“There are a couple of websites that I go to, that I trust the data being used, to look at the outcomes. If I felt the data were trustworthy, I would use the data. I’ve looked up where the ProPublica data has come from.”
ProPublica; Hospital Compare	Not specific to operation of interest	“I don’t think of [websites with outcomes data] as decision aids; they rank hospitals on their outcomes, they rank surgeons on their outcomes—not always on the surgery you’re interested in, but you can often find the surgeon.”
Hospital Compare	Difficult to use; unclear data quality	“If there were something that I knew was unbiased and easy to access, I would use it. I know there are websites that have gradations and I know that CMS [Centers for Medicare & Medicaid Services] Medicare does publish data on hospitals and on individual surgeons, but I find that so cumbersome to find that I’ve never been convinced that it’s any better than my local knowledge.”
Internet	Not specific to operation of interest	“I don’t have any data access except Googling it or just going to the internet regarding that doctor. I know there are some websites we can check, but regarding a certain website for a surgeon for colorectal cancer—I haven’t seen anything like that.”
Google	Difficult to find	“I do not look at that data [professionally], but in my personal life, when there’s a family member who is going to look at a surgeon, I do look at that [data]. I would love to use that data.…It’s hard to find. I Googled it and was trying to find it for hip surgeons in the region for my uncle. [The data] would affect my prescribing and my referral patterns, but it is hard to incorporate.”
Cancer center website	None noted	“…what I sometimes do for cancers that are more obscure is I will look at a particular cancer center’s website and I will look for a specialist surgeon that mentions added training in that area.”

^a^ Nonstandardized abbreviations have been expanded. Institutional names have been replaced with generic terms.

Primary care physicians cited the convenience of in-network referrals as a factor associated with surgeon and hospital selection. Convenience was segmented by physician and physician-perceived patient considerations. Primary care physicians considered within-network advantages in care coordination such as use of a common electronic health record for immediate communication and medical record access. As for perceived patient considerations, PCPs considered compatibility with insurance, availability of social support, and location of the clinician.

## Discussion

In keeping with common practice patterns noted in the literature,^13^ participating PCPs reported frequent use of DSTs to inform medication selection. In contrast, we show for the first time, to our knowledge, that DSTs are not used when making surgical referrals. According to the participants, PCPs rely on their professional experiences and training, perceived surgeon and hospital quality, and convenience to differentiate between specific surgeons or hospitals.

Similar to other studies, we found that PCPs strongly consider surgeon and hospital “quality” when making surgical recommendations.^2,14^ However, when probed on how they determine surgeon and hospital quality, PCPs reported using anecdotal interactions due to existing data limitations. This finding is problematic because surgeon or hospital reputation and measured quality are poorly correlated.^15^

One solution to improve the surgical referral process is to develop DSTs similar to those used for medication selection. To be effective, DSTs for surgical referrals will have to overcome PCP concerns regarding data reliability, availability, and accessibility. Given the variation in surgical outcomes across hospitals and surgeons,^4^ data-driven referral practices may improve surgical outcomes by encouraging PCPs to refer to objectively higher-quality clinicians.

### Limitations

This study has some limitations. First, this study captured the perspectives of PCPs in the mid-Atlantic region of the US and may not be generalizable to other regions. Second, the voluntary sample lacked Black and Hispanic PCPs; thus, it was not fully representative of the national PCP population.

## Conclusions

This qualitative study suggests that PCPs’ common practice of using DSTs for medication selection may be transferrable to the surgical referral process. In the absence of objective data, PCPs rely on professional experiences, personal beliefs about quality, and convenience to guide surgical recommendations. Surgical outcomes data with tools to aid in decision-making may disrupt current physician referral practices and improve access to high-quality surgical care.

## Notes

Supplement 1. **eTable 1.** Pre-Scheduling Survey **eTable 2.** Interview Guide

Supplement 2.
Data Sharing Statement
