# Supplementary material for: Factors Associated With Primary Care Physician Decision-making When Making Medication Recommendations vs Surgical Referrals
Source: JAMA Netw Open. 2023 Feb 15;6(2):e2256086. doi: 10.1001/jamanetworkopen.2022.56086 (PMC9932841; doi:10.1001/jamanetworkopen.2022.56086)
Supplement: Supplement 1. — eTable 1. Pre-Scheduling Survey eTable 2. Interview Guide [file jamanetwopen-e2256086-s001.pdf]

Supplementary Online Content

Naik A, Syvyk S, Tong J, et al. Factors associated with primary care physician decision-making when making medication recommendations vs surgical referrals. *JAMA Netw Open.* 2023;6(2):e2256086. doi:10.1001/jamanetworkopen.2022.56086

eTable 1. Pre-Scheduling Survey
eTable 2. Interview Guide

This supplementary material has been provided by the authors to give readers additional information about their work.

© 2023 Naik A et al. *JAMA Network Open.*

eTable 1. Pre-Scheduling Survey
Sent to primary care providers in the University of Pennsylvania Primary Care Network by the Medical Director of the Primary Care Service Line (MP) in April 2021.

Physician Practice Characteristics

1. How often do you see patients? ____
2. How many partners do you have in your practice? 0, 1-3, 4-10, 10+
3. How would you characterize your practice?
    a. Minority-serving yes/no
    b. Geriatric yes/no
    c. Urban/non-urban
    d. University-affiliated yes/no
    e. Independent or hospital-affiliated
4. What is the zip code of your dominant practice location? (i.e., The practice where you spend the majority of your clinical time)
5. How would you describe your age group? 30-55, 56-69, 70+ (optional)
6. What is your sex at birth? (optional)
7. What is your gender? (optional)
8. Are you Hispanic/Latino? yes/no (optional)
9. How do you define your race? __________________________ (optional)
10. How many years have you been in practice? 0-10y, 11-30y, 30+
11. Do you use an electronic health record? Yes/no
12. What is your specialty? Internal Medicine, Family Practice, Other ___________________
13. If you would like to do xyz, then email confirmation X 2
14. Phone number

© 2023 Naik A et al. *JAMA Network Open.*

eTable 2. Interview Guide

FREELIST DATA COLLECTION-PATIENT

Thank you for agreeing to participate in this interview. We are interested in learning from you about what it's like to **refer patients for surgical care for colorectal cancer**. As a primary care physician, you are often the **gatekeeper to surgical care** and you can **help us to understand** the current referral process and **define a new approach for data-driven referrals**.
For **this interview** we are going to **make some lists of things** and then **ask several open-ended questions.**

Part 1: Free listing

Let's begin the free listing portion by practicing making lists.
When you make the lists, please try to limit your answers to one word, if possible. I will write them down in the order that you give them to me. **There are no right or wrong answers.** We are just interested in what you think.
Okay, **we are now ready to begin**: For this practice list, please try to list all of your **favorite types of fruit**.

1	11
2	12
3	13
4	14
5	15
6	16
7	17
8	18
9	19
10	20

SKIP If Using Next Question: Next, I would like to ask you to list all of the factors that you consider when recommending a surgeon for colorectal surgery for cancer to your patients. Please try to give one- or two-word answers.

© 2023 Naik A et al. *JAMA Network Open.*

➔ (**Probe:** Perhaps, think about the last time that you referred someone for a cancer operation. Which factors came into your mind as you decided **who** to recommend?)

1	11
2	12
3	13
4	14
5	15
6	16
7	17
8	18
9	19
10	20

SKIP If Previous Used: Next, I would like to ask you to <u>list all of the factors that you consider when recommending a hospital for colorectal surgery for cancer to your patients</u>. Please try to give one- or two-word answers.

➔ (Probe: Think about the last time that you referred someone for a cancer operation. Which factors came into your mind as you decided **where** to recommend?)

1	11
2	12
3	13
4	14
5	15
6	16
7	17
8	18
9	19
10	20

© 2023 Naik A et al. *JAMA Network Open.*

Now, I would like to ask you to think about the last time that you **<u>prescribed a statin for a patient</u>**. (Pause).
What **<u>information or decision aids</u>** did you use when **<u>recommending a specific medication</u>** to your patients?

1	11
2	12
3	13
4	14
5	15
6	16
7	17
8	18
9	19
10	20

Now, Please think about the last time that you referred a patient for a ***<u>colorectal cancer operation.</u>***
Please list the source of the information or decision aids that you use to guide recommendations on specific providers?

1	11
2	12
3	13
4	14
5	15
6	16
7	17
8	18

© 2023 Naik A et al. *JAMA Network Open.*

9	19
10	20

That **concludes** the FREELISTING portion of this interview. Now, I would like to switch gears once more and ask you a few more **pointed questions** about the surgical referral process.

Part 2: Semi-structured questions

1. **USE OF DATA** What are all of the things you consider when you make a referral for someone with colorectal cancer?

No mention of Data – ASK: "You did not mention data. Can you please tell me about any time you might have used data?"

If answer to question includes data - Follow-up with …

A. ➔ "Think about last time you used data on surgical outcomes to inform referrals. Please tell me about that."

(Probe: B. ➔ Please describe your initial opinions of the available data. What role does trust play in this process?)

Probe into this. **Why do they trust it or not trust it? Do they think it should have a role in referrals? What is their role in knowing the performance metrics of the surgeons that they refer to?**

2. **QUALITY OF SURGEON** Describe your-thoughts on how you would define a high-quality surgeon.

If answer to question includes skill - Follow-up with …

→ **Probe:** What is your understanding of/ how do you define surgical skill or access information on it?

3. **AVAILABLE DATA** If you could access **valid and reliable information** on surgeon or hospital performance, **how** would you use it?

Probes:

→ All Factors: What would **help you to use** it? What would **prevent you** from using it?

→ Location: "Where would you like to access information on surgeon or hospital performance specific to your patient?"

→ Process: "**At what point** would you use it?" or "**Who in your practice** would use it?"

→ Patient: How do you **handle patient preferences** for treatment location?

4. **ACCESS** What do you do when the **best** hospital or surgeon is **outside of your health system**?

What would help you to make a **referral outside of your system** or network if you felt that was **in** your patient's best interest?

What **affects your ability to refer outside** of your system or network?

That concludes the interview.

Thank you!

© 2023 Naik A et al. *JAMA Network Open.*
