# Supplementary material for: Factors Associated With Primary Care Physician Decision-making When Making Medication Recommendations vs Surgical Referrals
Source: JAMA Netw Open. 2023 Feb 15;6(2):e2256086. doi: 10.1001/jamanetworkopen.2022.56086 (PMC9932841; doi:10.1001/jamanetworkopen.2022.56086)
Supplement: Supplement 2. — Data Sharing Statement [file jamanetwopen-e2256086-s002.pdf]

# Data Sharing Statement

Naik. Factors Associated With Primary Care Physician Decision-making When Making Medication Recommendations vs Surgical Referrals. *JAMA Netw Open*. Published February 15, 2023. doi:10.1001/jamanetworkopen.2022.56086

**Data**
**Data available:** No
